# Rapid Emergence and Evolution of Staphylococcus aureus Clones Harboring *fusC*-Containing Staphylococcal Cassette Chromosome Elements

**DOI:** 10.1128/AAC.03020-15

**Published:** 2016-03-25

**Authors:** Sarah L. Baines, Benjamin P. Howden, Helen Heffernan, Timothy P. Stinear, Glen P. Carter, Torsten Seemann, Jason C. Kwong, Stephen R. Ritchie, Deborah A. Williamson

**Affiliations:** a Doherty Applied Microbial Genomics, Department of Microbiology & Immunology, The University of Melbourne at The Doherty Institute for Infection and Immunity, Melbourne, Australia; b Microbiological Diagnostic Unit Public Health Laboratory, Department of Microbiology & Immunology, The University of Melbourne at The Doherty Institute for Infection and Immunity, Melbourne, Australia; c Infectious Diseases Department, Austin Health, Melbourne, Australia; d Institute of Environmental Science and Research, Wellington, New Zealand; e Victorian Life Sciences Computation Initiative, The University of Melbourne, Melbourne, Australia; f School of Medical Sciences, University of Auckland, Auckland, New Zealand

## Abstract

The prevalence of fusidic acid (FA) resistance among Staphylococcus aureus strains in New Zealand (NZ) is among the highest reported globally, with a recent study describing a resistance rate of approximately 28%. Three FA-resistant S. aureus clones (ST5 MRSA, ST1 MSSA, and ST1 MRSA) have emerged over the past decade and now predominate in NZ, and in all three clones FA resistance is mediated by the *fusC* gene. In particular, ST5 MRSA has rapidly become the dominant MRSA clone in NZ, although the origin of FA-resistant ST5 MRSA has not been explored, and the genetic context of *fusC* in FA-resistant NZ isolates is unknown. To better understand the rapid emergence of FA-resistant S. aureus, we used population-based comparative genomics to characterize a collection of FA-resistant and FA-susceptible isolates from NZ. FA-resistant NZ ST5 MRSA displayed minimal genetic diversity and represented a phylogenetically distinct clade within a global population model of clonal complex 5 (CC5) S. aureus. In all lineages, *fusC* was invariably located within staphylococcal cassette chromosome (SCC) elements, suggesting that SCC-mediated horizontal transfer is the primary mechanism of *fusC* dissemination. The genotypic association of *fusC* with *mecA* has important implications for the emergence of MRSA clones in populations with high usage of fusidic acid. In addition, we found that *fusC* was colocated with a recently described virulence factor (*tirS*) in dominant NZ S. aureus clones, suggesting a fitness advantage. This study points to the likely molecular mechanisms responsible for the successful emergence and spread of FA-resistant S. aureus.

## INTRODUCTION

The commonest clinical manifestation of Staphylococcus aureus disease is skin and soft-tissue infection (SSTI). In most cases, S. aureus SSTI is diagnosed and managed in the primary care setting, and depending on the clinical manifestation, treatment may involve the administration of a topical antimicrobial agent, such as fusidic acid (FA) (1). FA is a topical and systemic antimicrobial and is active against several Gram-positive organisms. Recently licensed in the United States, FA has been used extensively in several settings for the topical treatment of superficial S. aureus SSTI (2). We have recently described a significant increase in topical FA use in New Zealand (NZ), concurrent with an increase in FA resistance rates in S. aureus, from 17% in 1999 to 28% in 2013 (2). This increase in resistance was associated with the emergence of three specific S. aureus clones: multilocus sequence type 5 (ST5) methicillin-resistant S. aureus (MRSA), ST1 methicillin-susceptible S. aureus (MSSA), and ST1 MRSA. In particular, an ST5 MRSA clone (colloquially known as AK3 ST5 MRSA) has emerged over the past decade to become the dominant community- and hospital-associated MRSA clone in NZ (3). First reported in 2005, AK3 ST5 MRSA strains harbor a type IV staphylococcal cassette chromosome (SCC) *mec* and, in addition to being resistant to penicillin and oxacillin, generally are resistant to FA, with one recent study describing an FA resistance rate of 89% in this clone (2). To date, however, the origin and evolutionary history of AK3 ST5 MRSA has not been determined, and it is unknown whether this clone has emerged from locally circulating ST5 MSSA or, alternatively, has been imported into NZ with subsequent regional transmission.

In all three clones (AK3 ST5 MRSA, ST1 MSSA, and ST1 MRSA), FA resistance is associated with the presence of the *fusC* gene (2). However, the origin and genetic context of the *fusC* resistance locus has not been investigated previously in S. aureus from NZ. To date, only a few studies have previously described the genetic context of *fusC* among small numbers of S. aureus lineages (4–7). Furthermore, the extent to which common mobile genetic elements are shared among *fusC*-harboring S. aureus clones globally has not been investigated previously but could provide important insights into the mechanisms of emerging resistance to topical antimicrobials across different S. aureus lineages.

Accordingly, the objectives of this study were to (i) investigate the evolution of the AK3 ST5 MRSA clone in NZ, (ii) characterize the genetic context of the *fusC* gene in dominant FA-resistant clones in NZ, namely, AK3 ST5 MRSA, ST1 MSSA, and ST1 MRSA, and (iii) compare these to international *fusC*-harboring isolates of S. aureus to better understand the rapid emergence of FA-resistant S. aureus.

## MATERIALS AND METHODS

### Bacterial isolates.

A total of 46 isolates of S. aureus recovered in NZ were used in this study. These isolates were sourced from (i) national surveys of MRSA carried out between 2005 and 2013 (*n* = 34) (https://surv.esr.cri.nz/antimicrobial/mrsa_annual.php) and (ii) a contemporary molecular epidemiological survey of S. aureus carried out in Auckland, NZ, in 2013 (*n* = 12) (2). Between two and six representatives of clonal complex 5 (CC5) were selected from each survey year, and six representatives of both ST5 and ST1 were selected from the contemporary molecular epidemiological survey. This collection additionally was supplemented with the fully assembled genomes of 11 S. aureus strains belonging to CC5 (8–16), the CC1 strain MSSA476 (4), and the genome sequence reads of 12 FA-resistant S. aureus strains from a recent study in the United Kingdom (UK) (6). All CC5 S. aureus strains from this study were included, as well as a single representative for each SCC element described for other S. aureus lineages. Further information for each isolate is available in Table S1 in the supplemental material.

### Genome sequencing.

Genomic DNA was extracted using the DNeasy blood and tissue extraction kit (Qiagen), and DNA libraries were created using the Nextera XT DNA preparation kit (Illumina). Sequencing was performed on either the HiSeq platform (Illumina) with 2- by 100-bp paired-end chemistry or the MiSeq platform (Illumina) with 2- by 250-bp paired-end chemistry. S. aureus isolate NZAK3 was sequenced on the Pacific Biosciences RS-II platform.

### Genome assembly of NZAK3.

The complete genome assembly of S. aureus reference strain NZAK3 was performed using the SMRT analysis system v2.3.0.140936 (Pacific Biosciences). Raw sequence data were *de novo* assembled using the HGAP3 protocol, with a minimum seed read length of 5,000, genome size of 3 Mb, target coverage of 10, and overlapper error rate of 0.04. Polished contigs were further error corrected using Quiver v1. The read alignments were visually assessed, and one rRNA region was found to have poor support; subsequently, the contig was cut at this region. The resulting contigs then were ordered against S. aureus N315 (8) using Mauve (17) and joined in Geneious v8.1.5 (Biomatters); the altered assembly around the rRNA locus was confirmed with long-range PCR. The final assembly was checked using BridgeMapper v1 in the SMRT analysis system, and the consensus sequence was corrected with short-read Illumina data using Snippy v2.5 (https://github.com/tseemann/snippy). The final sequences were annotated using Prokka v1.11 (18), and *in silico* multilocus sequence typing (MLST) was performed using MLST v1.2 (https://github.com/tseemann/mlst). Sequence data for NZAK3 have been deposited in the European Nucleotide Archive (ENA) under primary accession PRJEB12333.

### Phylogenetic analysis.

The genomes of the 11 assembled CC5 S. aureus strains first were shredded into 100-bp pseudoreads with 50× coverage using Nesoni v0.130 (https://github.com/Victorian-Bioinformatics-Consortium/nesoni). The sequence reads of all CC5 S. aureus strains then were mapped to reference strain NZAK3, and single-nucleotide polymorphisms (SNPs) were detected using Snippy v2.5 with a minimum coverage depth of 10 and base call stringency of 90%. A maximum likelihood (ML) phylogenetic tree was constructed from core genome SNPs using PhyML v3.0 (19) run under a general time-reversible model with 1,000 bootstrap replicates.

### Analysis of the genetic context of *fusC* and alignment of *fusC*-containing SCC elements.

The genome sequence reads of all unassembled isolates were *de novo* assembled using SPAdes v3.6.1 (20). The program contig-puller (https://github.com/kwongj/contig-puller) then was used to identify, extract, and annotate contigs that contained a *fusC* gene with 99% sequence identity to that present in S. aureus MSSA476 (GenBank accession number BX571857, locus tag SAS0043). These contigs were manually examined in Geneious v8.1.5 (Biomatters). Sequence alignments were performed using ClustalW v2.1 (21) and the Artemis comparison tool v16.0.11 (22).

### BioProject sequence accession number.

All sequence data are available at the ENA under primary accession no. PRJEB12333.

## RESULTS AND DISCUSSION

### Complete genome sequence of S. aureus NZAK3.

To characterize AK3 ST5 MRSA and provide a closely related reference genome for subsequent phylogenetic analysis, the first reported strain of AK3 ST5 MRSA was sequenced and completely assembled using PacBio sequencing. This strain, subsequently referred to as NZAK3, was isolated in Auckland, NZ, in 2005 from a patient with SSTI. To our knowledge, this is the earliest isolation of this FA-resistant AK3 ST5 MRSA clone globally.

The genome of NZAK3 comprises a circular chromosome spanning 2,831,681 bp (GC content of 32.8%) and a single circular plasmid (pNZAK3) spanning 25,689 bp (GC content of 29.5%). A total of 2,607 protein-coding regions (CDS) were identified in the chromosome, and 30 CDS were identified in pNZAK3. The chromosome contained five rRNA operons and 62 tRNA genes. *In silico* MLST confirmed that this isolate belonged to ST5.

**FIG 1:**
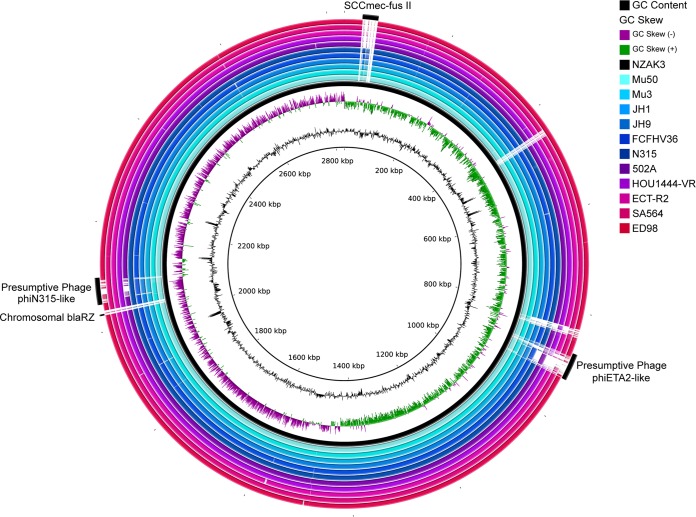
Sequence comparison of all complete genomes of clonal complex 5 (CC5) S. aureus strains. A BLAST-based comparison of all complete CC5 S. aureus genomes against representative FA-resistant strain NZAK3, visualized with BRIG, illustrates a high level of sequence conservation among members of the CC5 lineage. Genomic regions in NZAK3 that were variably present in other CC5 S. aureus strains include the SCC*mec-fus* II mobile element, a chromosomal copy of the *blaRZ* locus, and two complete phage regions, which were identified using PHAST as a presumptive phiETA2 (NC_008798) and phiN315 (NC_004740).

Comparison of the chromosome of NZAK3 to all other completely assembled S. aureus genomes belonging to CC5 (*n* = 11) illustrated a high level of sequence conservation among members of this lineage (Fig. 1). The accessory CC5 genome consisted primarily of mobile elements, specifically the SCC*mec* locus, a chromosomal copy of *blaZ*, and two complete prophages. These prophages were presumptively identified as phiETA2 (GenBank accession no. NC_008798) and phiN315 (GenBank accession no. NC_004740) based on a predominance of high-identity BLAST matches to each of these elements. The plasmid pNZAK3 was mostly unique, with only 45% (11,588/25,689 bp) of the sequence matching a previously published plasmid sequence with 99% DNA identity (plasmid SAP059A; GenBank accession no. GQ900480.1). The remaining region in pNZAK3 contained a cadmium resistance locus but no other known resistance or virulence determinants.

### Population structure and evolution of AK3 ST5 MRSA.

To characterize the population structure of the AK3 clone and investigate its evolutionary origins, 39 isolates of S. aureus recovered in NZ and belonging to CC5 underwent whole-genome sequencing. To maximize temporal and geographic diversity within the collection, between two and six isolates were selected from each year between 2006 and 2013 and from different regions in NZ (detailed in Materials and Methods). This collection also was supplemented with the 11 fully assembled CC5 genome sequences described above and the genome sequence reads of eight CC5 MRSA isolates from a recent study of FA-resistant S. aureus in the United Kingdom (6). Details of these isolates are available in Table S1 in the supplemental material.

**FIG 2:**
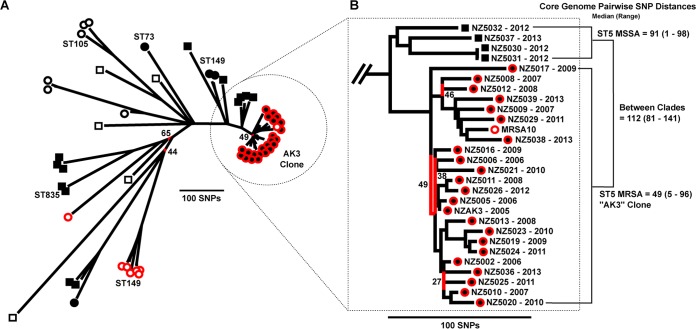
Population structure of fusidic acid-resistant CC5 S. aureus. (A) The maximum likelihood tree, constructed from core genome SNPs, illustrates the population structure of a representative global population of CC5 S. aureus strains. Terminal branch symbols indicate the location from which isolates were recovered and both methicillin and fusidic acid resistance phenotypes. Symbol shape: circle, MRSA; square, MSSA. Inner symbol color: black, recovered in New Zealand; white, recovered outside New Zealand. Outer symbol color: red, fusidic acid resistant; black, fusidic acid susceptible. (B) Enlarged view of the population structure for the AK3 ST5 MRSA clade and a locally circulating ST5 MSSA clade. The core genome pairwise SNP distances, calculated from the SNP matrix used to construct this tree, indicates the median and range of SNPs that separate any two isolates within the AK3 ST5 MRSA clade, the ST5 MSSA clade, and between the two clades. In both panels A and B, branches with less than 70% bootstrap support are colored red, and the exact support percentage is provided.

Mapping of the genome sequence data of these 58 CC5 S. aureus strains to reference strain NZAK3 identified a total of 14,584 SNPs, of which 4,233 were present within the core genome (87.3% of reference strain NZAK3). The 4,233 core genome SNPs were used to construct an ML phylogenetic tree (Fig. 2).

This collection of CC5 S. aureus isolates represented five sequence types: ST5, ST73, ST105, ST149, and ST835 (Fig. 2). Isolates of the same ST typically grouped together in ST-specific clades, the only exception being ST5, which represented multiple distinct clades and singletons. In general, isolates from NZ clustered together, and only one isolate, recovered outside NZ, was found to cluster with an NZ-specific clade (Fig. 2). This NZ clade represented the AK3 ST5 MRSA clone, and all FA-resistant CC5 isolates recovered from NZ resided within this clade. The median core genome pairwise SNP distance between two isolates in this clade was only 49 SNPs, with a minimum distance of five SNPs and a maximum distance of 96 SNPs, highlighting the extremely restricted genetic diversity of FA-resistant ST5 MRSA in NZ. The single international isolate to cluster with the AK3 ST5 MRSA clade was an FA-resistant MRSA isolate (MRSA10) recovered in the UK during 2011 to 2012 (6). MRSA10 had a median core genome pairwise SNP distance of only 59 SNPs to other AK3 isolates. Interestingly, the other CC5 FA-resistant isolates from the United Kingdom formed a single ST149 clade (*n* = 6) and one ST5 singleton. Taken together, these findings suggest that AK3 ST5 MRSA forms a genetically distinct clone within ST5, as indicated by the genetic distance between the AK3 clade and representative international ST5 isolates. Furthermore, the location of UK isolate MRSA10 in the AK3 clade suggests potential recent intercontinental transmission between these two countries.

The inclusion of FA-susceptible isolates in this population model also enabled an investigation into the origin of the AK3 ST5 MRSA clone. The phylogenetic proximity of the AK3 ST5 MRSA clade to four ST5 MSSA isolates recovered from NZ suggests that this clone emerged from locally circulating MSSA rather than being imported from overseas (Fig. 2), as has been described for ST5 MRSA in other settings (23). Furthermore, locally circulating ST149 S. aureus strains (belonging to CC5) also were found to be more similar at a core genome level to AK3 ST5 MRSA than to representative international lineages of ST5. The hypothesis that AK3 ST5 MRSA emerged from local ST5 MSSA also was supported by the absence of any remnants of an SCC*mec* element in the four NZ ST5 MSSA strains. As described below, the *fusC* gene was present in all FA-resistant AK3 ST5 MRSA isolates as part of a chimeric SCC*mec*-SCC mobile element integrated into the chromosome at *orfX*. The inspection of the region surrounding *orfX* found it to be intact in all four ST5 MSSA isolates (see Fig. S1 in the supplemental material), suggesting that these isolates had not previously carried an SCC element in their ancestral genome. Therefore, it is possible that the acquisition of *fusC* in the AK3 ST5 MRSA clone occurred more recently, following its divergence from locally circulating ST5 MSSA, although this hypothesis should be explored further in the framework of a larger sample of international ST5 MSSA.

### Genetic context of *fusC* in ST1 and ST5 S. aureus isolates.

In order to elucidate the genetic context of the *fusC* gene among NZ lineages of S. aureus and to compare it to other described CC5 and ST1 *fusC*-containing SCC elements, the genomes of *fusC*-harboring isolates were *de novo* assembled. These included 23 isolates belonging to the AK3 ST5 MRSA clade and six *fusC*-harboring ST1 S. aureus isolates recovered in New Zealand. In addition, six representative CC5 and ST1 S. aureus isolates from published studies were included. These isolates contained previously described *fusC*-containing mobile elements, including SCC_476_ in MSSA476 (ST1) (4), and previously described elements from a recent study (6), namely, the composite elements SCC*mec*IVa-SCC_476_ in isolate MRSA2 (ST1 MRSA), SCC_149_-SCC*mec*IVa in isolate MRSA17 (ST149 MRSA), and the chimeric elements SCC*mec-fus*I in isolate MRSA3 (ST1 MRSA), SCC*mec-fus*II in isolate MRSA10 (ST5 MRSA), and SCC*mec-fus*III in isolate MRSA7 (ST1 MRSA) (see Table S1 in the supplemental material). In a previous study by Ellington et al. (6), the chimeric elements SCC*mec-fus*II (29.1 kb) and SCC*mec-fus*III (25.4 kb) were reported to have been identified in isolates MRSA7 (ENA accession number ERR108061) and MRSA10 (ENA accession number ERR108040), respectively. However, we found this to be the reverse, with the sequence reads available for isolate MRSA7 containing the shorter SCC*mec-fus*III element and for MRSA10 containing the longer SCC*mec-fus*II element. Subsequently, we refer to these chimeric elements with the annotations previously reported (6) but with the isolate names reversed.

**FIG 3:**
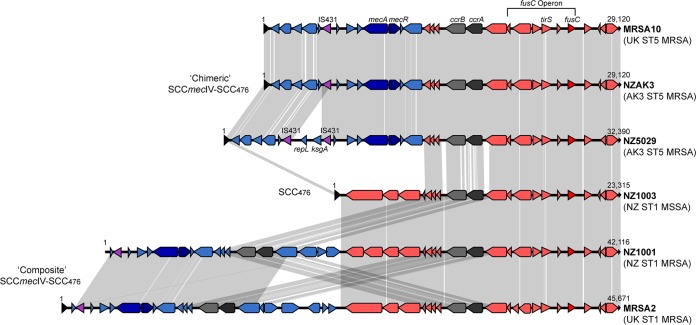
Comparison of *fusC*-containing staphylococcal cassette chromosome elements in ST1 and ST5 lineages of S. aureus. Schematic diagram illustrating the genetic organization of the SCC 476 (SCC_476_) element in ST1 MSSA (represented by strain NZ1003), the chimeric SCC*mec*IV-SCC_476_ element in ST5 MRSA (strain NZAK3), and the composite SCC*mec*-SCC_476_ element in ST1 MRSA (strain NZ1001). The transposon-containing region within the chimeric SCC*mec*IV-SCC_476_ element in strain NZ5029 also is illustrated. The SCC*mec* elements from MRSA2 and MRSA10 from a previous study (6) are included for comparison. Blue or purple arrows indicate sequences present within SCC*mec* regions. The direction of the arrows indicates the direction of transcription for open reading frames. Only coding sequences of >200 bp are shown. Gray shaded areas represent regions that share >99% nucleotide sequence identity.

Among ST5 MRSA isolates, reference strain NZAK3 contained a chimeric SCC*mec*IV-SCC_476_ element spanning 29,120 bp that displayed 99.9% nucleotide sequence to the SCC*mec-fus*II element identified in isolate MRSA10 from the previous UK study (6) (Fig. 3). This finding further supports the likely intercontinental spread of this clone, as suggested by the population model. The examination of the draft assemblies of the other 23 AK3 ST5 MRSA isolates found that they all carried *fusC* as part of a chimeric SCC*mec*-SCC_476_ element, although in 15 isolates this element was fragmented and located across two contigs. The longest fragment ranged in length from 78% to 87% of the full length of the *fusC*-containing chimeric SCC*mec*IV-SCC_476_ and displayed >99.7% nucleotide sequence identity to the chimeric SCC*mec*IV-SCC_476_ element found in NZAK3. Of the eight isolates with a complete SCC*mec*-SCC_476_ element, seven displayed >99.9% nucleotide sequence identity to the chimeric SCC*mec*IV-SCC_476_ element in NZAK3. The single AK3 ST5 MRSA isolate with a different chimeric SCC*mec*IV-SCC_476_ element was isolate NZ5029, which displayed only 89.9% nucleotide identity to the chimeric SCC*mec*IV-SCC_476_ element in NZAK3. A visual assessment of this alignment identified that the chimeric SCC*mec*IV-SCC_476_ element in NZ5029 contained a previously unreported transposon insertion within the SCC*mec*IV portion upstream of *mecA* (Fig. 3). This transposon was flanked by two copies of IS*431*, and the intervening region encoded two genes annotated as a *repL* (firmicute plasmid replication protein) and *ksgA* (dimethyladenosine transferase). This insertion was exclusive to this strain, as a nucleotide BLAST search for the *ksgA* gene returned no hits among the other AK3 ST5 MRSA isolates.

Importantly, the high level of nucleotide identity between *fusC*-containing SCC elements in AK3 ST5 MRSA isolates supports the hypothesis that the AK3 ST5 MRSA clone emerged following a single acquisition of the chimeric *fusC*-containing SCC*mec*IV-SCC_476_ element with subsequent clonal expansion. Furthermore, the colocation of *fusC* in the same mobile element as *mecA* is of particular concern in NZ, where the extensive use of topical FA in NZ appears not only to be selecting for FA-resistant S. aureus but also coselecting for MRSA. Therefore, the genotypic association of *fusC* with *mecA* has important implications for the emergence of MRSA clones in populations with high usage of fusidic acid.

In addition to ST5 MRSA, the two other dominant FA-resistant S. aureus lineages identified in NZ were ST1 MSSA and ST1 MRSA (2). To determine the genetic context of the *fusC* gene in these clones, six representative ST1 isolates were sequenced. Of these six isolates, five were MSSA and all carried *fusC* as part of an SCC element that displayed >99.8% nucleotide sequence identity to the previously described 23.3-kb SCC_476_ element identified in strain MSSA476 (4). In a recent survey, FA-resistant ST1 MSSA was the dominant MSSA clone circulating in the NZ community, accounting for 15% of all MSSA strains (2). This further supports the notion that high population use of FA has selected for dominant FA-resistant clones, resulting in marked changes in the molecular epidemiology of S. aureus in NZ (2, 3). The single ST1 MRSA isolate contained a composite SCC*mec*-SCC_476_ element (42,116 bp) but had only 88.9% nucleotide sequence identity to the only previously described composite element in ST1 MRSA, the SCC*mec*IVa-SCC_476_ element previously identified in isolate MRSA2 (6) (Fig. 3).

A comparison of the four different SCC elements identified in ST5 and ST1 S. aureus isolates in NZ and the two genetically similar SCC elements identified in isolates from the United Kingdom is presented in Fig. 3. The *fusC* gene was located consistently within the joining (J1) region between the *ccrAB2* genes and the downstream direct-repeat (DR) region of an SCC_476_ type element. Of note, among these six SCC elements, there was an ∼5-kb region with >99% nucleotide sequence identity upstream of *fusC*, suggesting a common ancestral origin for this specific sequence. Moreover, within this region was a *tirS* gene encoding a TlR domain named TirS. Recent work has suggested that TirS contributes to bacterial virulence by interfering with TLR2 signaling pathways and enhancing bacterial survival within the host (24). It is possible that, in addition to the presence of the *fusC* gene, the presence of TirS also confers a selective advantage for these dominant clones, although it is presently unknown whether clinical infections caused by TirS-producing S. aureus strains are more severe than those of TirS-deficient isolates. However, it is of concern that, in addition to the presence of genes encoding antimicrobial resistance, there is also evidence of horizontal transfer of a colocated putative virulence factor.

### Association of *fusC* with SCC-type elements in diverse lineages of S. aureus.

In this study, we found that *fusC* was invariably present within an SCC element, with or without the *mecA* gene. This also was found to be the case for other published *fusC*-harboring sequences deposited in the GenBank database, including SCC elements from Ireland (5) and Taiwan (7). This finding suggests that, similar to *mecA* transmission, SCC-mediated horizontal gene transfer is the major mechanism of *fusC* dissemination. This is distinct from the mechanism of dissemination of the other major acquired FA resistance gene, *fusB*, which is transferred primarily on plasmids, such as pUB101 and pUB102 (25, 26) and p11819-97, among CC80 S. aureus strains in Europe (27).

Of further interest was our finding that, in all previously identified *fusC*-containing SCC elements, both in this study and in other published sequences (4–7), *fusC* was always present within a conserved ∼1.3-kb region, which shared one upstream putative CDS (Fig. 2). A BLASTX and Pfam search did not assign any functional roles for these putative proteins. However, given the consistent finding of this CDS in association with *fusC*, it is possible that this protein plays a role in the regulation or modulation of *fusC* transcription. Future work should attempt to elucidate the function of these proteins in relation to *fusC* expression.

### Conclusions.

In this study, we described the emergence and possible origins of fusidic acid-resistant AK3 ST5 MRSA, the most prevalent MRSA clone in NZ. On the basis of population-based comparative genomics, it is likely that this clone has emerged from locally circulating ST5 MSSA strains in New Zealand. The AK3 ST5 MRSA clone carries a chimeric SCC*mec*IV-SCC_476_ element harboring *fusC*, which appears to have been acquired once and maintained among members of this clone. The invariable presence of *fusC* within an SCC-type element indicates that SCC-mediated horizontal transfer is the primary mechanism for dissemination of *fusC*. The genotypic association of *fusC* with *mecA* has important implications for the emergence of MRSA in populations with high usage of fusidic acid, and the colocation of *fusC* with *tirS* may confer an additional fitness advantage. The finding of AK3 ST5 MRSA in the United Kingdom suggests intercontinental transmission, which should be of concern to other regions that use topical FA to treat superficial SSTI. This study has revealed the rapidity and process by which an S. aureus population evolved and expanded in response to a human therapeutic intervention, in this case the increased use of topical FA. In the face of these data and those of related studies, it is clear that all of our efforts to control bacterial infections with antibiotics, disinfectants, and even vaccines are being met by swift Darwinian responses. The use of genomics for the prospective surveillance of clinical bacterial populations will be a critical monitoring tool in an imminent postantibiotic era.

## Supplementary Material

Supplemental material

## References

[1] Stevens DL, Bisno AL, Chambers HF, Everett ED, Dellinger P, Goldstein EJ, Gorbach SL, Hirschmann JV, Kaplan EL, Montoya JG, Wade JC 2005 Practice guidelines for the diagnosis and management of skin and soft-tissue infections. Clin Infect Dis 41:1373–1406. doi:10.1086/497143. 16231249

[2] Williamson DA, Monecke S, Heffernan H, Ritchie SR, Roberts SA, Upton A, Thomas MG, Fraser JD 2014 High usage of topical fusidic acid and rapid clonal expansion of fusidic acid-resistant Staphylococcus aureus: a cautionary tale. Clin Infect Dis 59:1451–1454. doi:10.1093/cid/ciu658. 25139961

[3] Williamson DA, Roberts SA, Ritchie SR, Coombs GW, Fraser JD, Heffernan H 2013 Clinical and molecular epidemiology of methicillin-resistant Staphylococcus aureus in New Zealand: rapid emergence of sequence type 5 (ST5)-SCCmec-IV as the dominant community-associated MRSA clone. PLoS One 8:e62020. doi:10.1371/journal.pone.0062020. 23637953 PMC3636228

[4] Holden MT, Feil EJ, Lindsay JA, Peacock SJ, Day NP, Enright MC, Foster TJ, Moore CE, Hurst L, Atkin R, Barron A, Bason N, Bentley SD, Chillingworth C, Chillingworth T, Churcher C, Clark L, Corton C, Cronin A, Doggett J, Dowd L, Feltwell T, Hance Z, Harris B, Hauser H, Holroyd S, Jagels K, James KD, Lennard N, Line A, Mayes R, Moule S, Mungall K, Ormond D, Quail MA, Rabbinowitsch E, Rutherford K, Sanders M, Sharp S, Simmonds M, Stevens K, Whitehead S, Barrell BG, Spratt BG, Parkhill J 2004 Complete genomes of two clinical Staphylococcus aureus strains: evidence for the rapid evolution of virulence and drug resistance. Proc Natl Acad Sci U S A 101:9786–9791. doi:10.1073/pnas.0402521101. 15213324 PMC470752

[5] Kinnevey PM, Shore AC, Brennan GI, Sullivan DJ, Ehricht R, Monecke S, Slickers P, Coleman DC 2013 Emergence of sequence type 779 methicillin-resistant Staphylococcus aureus harboring a novel pseudo staphylococcal cassette chromosome mec (SCC*mec*)-SCC-SCCCRISPR composite element in Irish hospitals. Antimicrob Agents Chemother 57:524–531. doi:10.1128/AAC.01689-12. 23147725 PMC3535981

[6] Ellington MJ, Reuter S, Harris SR, Holden MT, Cartwright EJ, Greaves D, Gerver SM, Hope R, Brown NM, Török ME, Parkhill J, Köser CU, Peacock SJ 2015 Emergent and evolving antimicrobial resistance cassettes in community-associated fusidic acid and meticillin-resistant Staphylococcus aureus. Int J Antimicrob Agents 45:477–484. doi:10.1016/j.ijantimicag.2015.01.009. 25769787 PMC4415905

[7] Lin YT, Tsai JC, Chen HJ, Hung WC, Hsueh PR, Teng LJ 2014 A novel staphylococcal cassette chromosomal element, SCC*fusC*, carrying *fusC* and *speG* in fusidic acid-resistant methicillin-resistant Staphylococcus aureus. Antimicrob Agents Chemother 58:1224–1227. doi:10.1128/AAC.01772-13. 24277045 PMC3910889

[8] Kuroda M, Ohta T, Uchiyama I, Baba T, Yuzawa H, Kobayashi I, Cui L, Oguchi A, Aoki K, Nagai Y, Lian J, Ito T, Kanamori M, Matsumaru H, Maruyama A, Murakami H, Hosoyama A, Mizutani-Ui Y, Takahashi NK, Sawano T, Inoue R, Kaito C, Sekimizu K, Hirakawa H, Kuhara S, Goto S, Yabuzaki J, Kanehisa M, Yamashita A, Oshima K, Furuya K, Yoshino C, Shiba T, Hattori M, Ogasawara N, Hayashi H, Hiramatsu K 2001 Whole genome sequencing of meticillin-resistant Staphylococcus aureus. Lancet 357:1225–1240. doi:10.1016/S0140-6736(00)04403-2. 11418146

[9] Parker D, Narechania A, Sebra R, Deikus G, Larussa S, Ryan C, Smith H, Prince A, Mathema B, Ratner AJ, Kreiswirth B, Planet PJ 2014 Genome sequence of bacterial interference strain Staphylococcus aureus 502A. Genome Announc 2:e00284–14. 24723721 10.1128/genomeA.00284-14 PMC3983310

[10] Lindqvist M, Isaksson B, Grub C, Jonassen TO, Hallgren A 2012 Detection and characterisation of SCCmec remnants in multiresistant methicillin-susceptible Staphylococcus aureus causing a clonal outbreak in a Swedish county. Eur J Clin Microbiol Infect Dis 31:141–147. doi:10.1007/s10096-011-1286-y. 21590357

[11] Lowder BV, Guinane CM, Ben Zakour NL, Weinert LA, Conway-Morris A, Cartwright RA, Simpson AJ, Rambaut A, Nubel U, Fitzgerald JR 2009 Recent human-to-poultry host jump, adaptation, and pandemic spread of Staphylococcus aureus. Proc Natl Acad Sci U S A 106:19545–19550. doi:10.1073/pnas.0909285106. 19884497 PMC2780746

[12] Panesso D, Planet PJ, Diaz L, Hugonnet JE, Tran TT, Narechania A, Munita JM, Rincon S, Carvajal LP, Reyes J, Londono A, Smith H, Sebra R, Deikus G, Weinstock GM, Murray BE, Rossi F, Arthur M, Arias CA 2015 Methicillin-susceptible, vancomycin-resistant Staphylococcus aureus, Brazil. Emerg Infect Dis 21:1844–1848. doi:10.3201/eid2110.141914. 26402569 PMC4593430

[13] Mwangi MM, Wu SW, Zhou Y, Sieradzki K, de Lencastre H, Richardson P, Bruce D, Rubin E, Myers E, Siggia ED, Tomasz A 2007 Tracking the in vivo evolution of multidrug resistance in Staphylococcus aureus by whole-genome sequencing. Proc Natl Acad Sci U S A 104:9451–9456. doi:10.1073/pnas.0609839104. 17517606 PMC1890515

[14] Neoh HM, Cui L, Yuzawa H, Takeuchi F, Matsuo M, Hiramatsu K 2008 Mutated response regulator graR is responsible for phenotypic conversion of Staphylococcus aureus from heterogeneous vancomycin-intermediate resistance to vancomycin-intermediate resistance. Antimicrob Agents Chemother 52:45–53. doi:10.1128/AAC.00534-07. 17954695 PMC2223914

[15] Giraud C, Hausmann S, Lemeille S, Prados J, Redder P, Linder P 2015 The C-terminal region of the RNA helicase CshA is required for the interaction with the degradosome and turnover of bulk RNA in the opportunistic pathogen Staphylococcus aureus. RNA Biol 12:658–674. doi:10.1080/15476286.2015.1035505. 25997461 PMC4615653

[16] McCulloch JA, Silveira AC, Lima Moraes Ada C, Perez-Chaparro PJ, Ferreira Silva M, Almeida LM, d'Azevedo PA, Mamizuka EM 2015 Complete genome sequence of Staphylococcus aureus FCFHV36, a methicillin-resistant strain heterogeneously resistant to vancomycin. Genome Announc 3:e00893–15. 26272570 10.1128/genomeA.00893-15 PMC4536681

[17] Darling AC, Mau B, Blattner FR, Perna NT 2004 Mauve: multiple alignment of conserved genomic sequence with rearrangements. Genome Res 14:1394–1403. doi:10.1101/gr.2289704. 15231754 PMC442156

[18] Seemann T 2014 Prokka: rapid prokaryotic genome annotation. Bioinformatics 30:2068–2069. doi:10.1093/bioinformatics/btu153. 24642063

[19] Guindon S, Delsuc F, Dufayard JF, Gascuel O 2009 Estimating maximum likelihood phylogenies with PhyML. Methods Mol Biol 537:113–137. doi:10.1007/978-1-59745-251-9_6. 19378142

[20] Bankevich A, Nurk S, Antipov D, Gurevich AA, Dvorkin M, Kulikov AS, Lesin VM, Nikolenko SI, Pham S, Prjibelski AD, Pyshkin AV, Sirotkin AV, Vyahhi N, Tesler G, Alekseyev MA, Pevzner PA 2012 SPAdes: a new genome assembly algorithm and its applications to single-cell sequencing. J Comput Biol 19:455–477. doi:10.1089/cmb.2012.0021. 22506599 PMC3342519

[21] Larkin MA, Blackshields G, Brown NP, Chenna R, McGettigan PA, McWilliam H, Valentin F, Wallace IM, Wilm A, Lopez R, Thompson JD, Gibson TJ, Higgins DG 2007 Clustal W and Clustal X version 2.0. Bioinformatics 23:2947–2948. doi:10.1093/bioinformatics/btm404. 17846036

[22] Carver TJ, Rutherford KM, Berriman M, Rajandream MA, Barrell BG, Parkhill J 2005 ACT: the Artemis comparison tool. Bioinformatics 21:3422–3423. doi:10.1093/bioinformatics/bti553. 15976072

[23] Nubel U, Roumagnac P, Feldkamp M, Song JH, Ko KS, Huang YC, Coombs G, Ip M, Westh H, Skov R, Struelens MJ, Goering RV, Strommenger B, Weller A, Witte W, Achtman M 2008 Frequent emergence and limited geographic dispersal of methicillin-resistant Staphylococcus aureus. Proc Natl Acad Sci U S A 105:14130–14135. doi:10.1073/pnas.0804178105. 18772392 PMC2544590

[24] Askarian F, van Sorge NM, Sangvik M, Beasley FC, Henriksen JR, Sollid JU, van Strijp JA, Nizet V, Johannessen M 2014 A Staphylococcus aureus TIR domain protein virulence factor blocks TLR2-mediated NF-kappaB signaling. J Innate Immun 6:485–498. 24481289 10.1159/000357618 PMC4198549

[25] Monecke S, Slickers P, Ehricht R 2008 Assignment of Staphylococcus aureus isolates to clonal complexes based on microarray analysis and pattern recognition. FEMS Immunol Med Microbiol 53:237–251. doi:10.1111/j.1574-695X.2008.00426.x. 18507678

[26] O'Brien FG, Price C, Grubb WB, Gustafson JE 2002 Genetic characterization of the fusidic acid and cadmium resistance determinants of Staphylococcus aureus plasmid pUB101. J Antimicrob Chemother 50:313–321. doi:10.1093/jac/dkf153. 12205055

[27] Stegger M, Wirth T, Andersen PS, Skov RL, De Grassi A, Simoes PM, Tristan A, Petersen A, Aziz M, Kiil K, Cirkovic I, Udo EE, del Campo R, Vuopio-Varkila J, Ahmad N, Tokajian S, Peters G, Schaumburg F, Olsson-Liljequist B, Givskov M, Driebe EE, Vigh HE, Shittu A, Ramdani-Bougessa N, Rasigade JP, Price LB, Vandenesch F, Larsen AR, Laurent F 2014 Origin and evolution of European community-acquired methicillin-resistant Staphylococcus aureus. mBio 5:e01044-14. doi:10.1128/mBio.01044-14. 25161186 PMC4173770

