# Supplementary material for: Rapid Emergence and Evolution of Staphylococcus aureus Clones Harboring fusC-Containing Staphylococcal Cassette Chromosome Elements
Source: Antimicrob Agents Chemother. 2016 Mar 25;60(4):2359–65. doi: 10.1128/AAC.03020-15 (PMC4808225; doi:10.1128/AAC.03020-15)
Supplement: Supplemental material [file supp_60_4_2359__index.html]

Rapid Emergence and Evolution of Staphylococcus aureus Clones Harboring fusC-Containing Staphylococcal Cassette Chromosome Elements — Supplemental material 

# Rapid Emergence and Evolution of Staphylococcus aureus Clones Harboring *fusC*-Containing Staphylococcal Cassette Chromosome Elements

## Supplemental material

- Supplemental file 1 -

  Fig. S1

  PDF, 240K
- Supplemental file 2 -

  Table S1

  XLSX, 15K
