# Supplementary material for: Rapid Emergence and Evolution of Staphylococcus aureus Clones Harboring fusC-Containing Staphylococcal Cassette Chromosome Elements
Source: Antimicrob Agents Chemother. 2016 Mar 25;60(4):2359–65. doi: 10.1128/AAC.03020-15 (PMC4808225; doi:10.1128/AAC.03020-15)

Figure S1: Alignment of region surrounding orfX demonstrating the intact orfX gene in ST5 MSSA from New Zealand that are closely related to NZAK3.

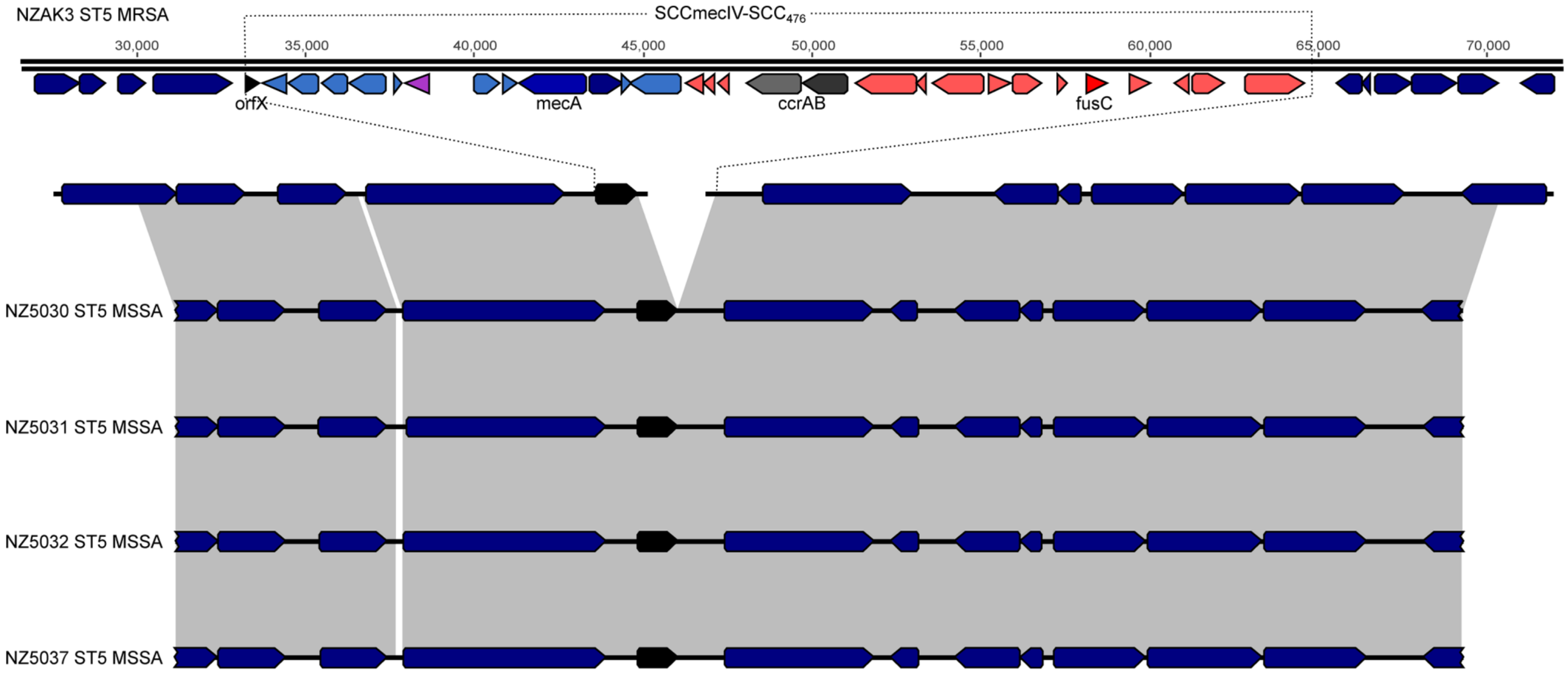

Supplement: Supplemental material [file AAC.03020-15_zac004165061so1.pdf]
